# Evaluating the implementation of cervical cancer screening programs in low-resource settings globally: a systematized review

**DOI:** 10.1007/s10552-020-01290-4

**Published:** 2020-03-17

**Authors:** J. Andrew Dykens, Jennifer S. Smith, Margaret Demment, E. Marshall, Tina Schuh, Karen Peters, Tracy Irwin, Scott McIntosh, Angela Sy, Timothy Dye

**Affiliations:** 1 University of Illinois at Chicago College of Medicine, Chicago, IL USA; 2 University of North Carolina School of Public Health, Chapel Hill, NC USA; 3 University of Rochester Department of Obstetrics and Gynecology, Rochester, NY USA; 4 University of Illinois at Chicago Institute for Health Research and Policy, Chicago, IL USA; 5 University of Washington Department of Obstetrics and Gynecology, Seattle, WA USA; 6 University of Rochester Department of Public Health Sciences, Rochester, NY USA; 7 University of Hawaii John A Burns School of Medicine, Honolulu, HI USA

**Keywords:** Cervical cancer screening, Implementation, Literature review, Prevention

## Abstract

**Purpose:**

Cervical cancer disproportionately burdens low-resource populations where access to quality screening services is limited. A greater understanding of sustainable approaches to implement cervical cancer screening services is needed.

**Methods:**

We conducted a systematized literature review of evaluations from cervical cancer screening programs implemented in resource-limited settings globally that included a formal evaluation and intention of program sustainment over time. We categorized the included studies using the continuum of implementation research framework which categorizes studies progressively from “implementation light” to more implementation intensive.

**Results:**

Fifty-one of 13,330 initially identified papers were reviewed with most study sites in low-resource settings of middle-income countries (94.1%) ,while 9.8% were in low-income countries. Across all studies, visual inspection of the cervix with acetic acid (58.8%) was the most prevalent screening method followed by cytology testing (39.2%). Demand-side (client and community) considerations were reported in 86.3% of the articles, while 68.6% focused scientific inquiry on the supply side (health service). Eighteen articles (35.3%) were categorized as “Informing Scale-up” along the continuum of implementation research.

**Conclusions:**

The number of cervical cancer screening implementation reports is limited globally, especially in low-income countries. The 18 papers we classified as Informing Scale-up provide critical insights for developing programs relevant to implementation outcomes. We recommend that program managers report lessons learnt to build collective implementation knowledge for cervical cancer screening services, globally.

**Electronic supplementary material:**

The online version of this article (10.1007/s10552-020-01290-4) contains supplementary material, which is available to authorized users.

## Introduction

Globally, there are over a half million new cases of cervical cancer yearly, with nearly 90% of these cases in least developed economies [1]. The United States (US) 2012 cervical cancer incidence rate was 8.1 per 100,000 women, while low- and middle- income countries (LMICs) had a collective rate of 15.7 [2]. Cervical cancer screening programs detect pre-cancerous lesions which can be treated with low-cost outpatient procedures, and if invasive cancers are caught early, successful treatments exist.

Cervical cancer disproportionately affects populations in resource-limited settings globally despite the variety of evidence-based screening options. Technological innovation and efficacy testing of health service interventions including various screening modalities [3, 4] have led to clear recommendations for improving cancer control among HPV vaccination programs and cervical cancer screening using locally appropriate methods [5–7]. Nonetheless, the judicious implementation of evidence-based cervical cancer screening programs remains inadequate, resulting in persistently elevated cervical cancer incidence and mortality rates, especially in resource-limited settings [8].

### Gaps in the literature

The *2011 WHO Prioritized Research Agenda for Prevention and Control of Non-communicable Disease* notes that while cancer screening services have been shown to be effective in high-income countries (HICs) [9–12] and HPV screening has been shown to reduce cervical cancer deaths in resource-limited settings such as rural India [13], there are few reports describing the implementation of successful and sustained cervical cancer screening programs in LMICs. Research that examines cervical cancer screening program barriers and facilitators within specific contexts and informs the adaptation of evidence-based interventions within these contexts is needed to ensure the successful implementation and sustainment of programs across various settings [6, 14].

In 2015, a scoping study of existing reviews on breast and cervical cancer in low- and middle- income countries concluded that current cervical cancer literature focuses primarily on prevention and detection, largely without implementation considerations [15]. An additional finding articulated that articles occasionally provide programmatic or policy recommendations that are beyond the context of their own studies’ findings. However, specific recommendations of the implementation methodologies relevant to issues of governance, systems development, workforce capacity, and person/ community centeredness for arriving at these conclusions are often lacking.

### Cervical cancer screening

Various evidence-based cervical cancer screening techniques have been developed, tested, and are appropriate for diverse contexts. High-resource settings often employ cytologic screening through Papanicolaou (Pap) smear with follow-up colposcopy and biopsy to identify early-stage dysplasia and pre-cancerous lesions. Cytologic screening can be resource heavy by requiring specialized specimen preservation and advanced technical expertise employing cyto-pathologists. Visual inspection methods can complement other screening modalities and provide adequate sensitivity and specificity to identify later-stage pre-cancers which can then be treated through cryotherapy freezing or loop electrosurgical excision procedure (LEEP), curative modalities commonly implemented alongside visual inspection screening services in low-resource settings. In addition, human papillomavirus (HPV) testing has the highest sensitivity for high-grade lesion detection and can be obtained through clinician-sampled or self-sampling techniques. HPV testing can be used for primary screening in conjunction with cytology or visual inspection for triage if the infrastructure exists. Despite the many screening modalities appropriate for a variety of contexts, there remains poor cervical cancer screening coverage, globally. Gakidou et al. reports a coverage rate of 36.9% globally and only 18.5% in least developed countries [16].

### Dissemination and implementation science for cancer research and health systems strengthening

This systematized review addresses the following questions: (1) “What published literature has reported implementation evaluations for cervical cancer screening programs in resource-limited settings, particularly those linked to sustainable systems (e.g., governmental) that will have a population-level impact?” and, (2) “What are the reported program implementation-relevant contextual findings applicable to guide the adaptation of cervical cancer screening programs into a wide range of settings to support cervical cancer prevention and control strengthening efforts worldwide?.”

By applying the “Continuum of Implementation Research” framework (see Table 4 for definitions and examples) to papers included within this review, our intent is to highlight how the existing catalog of cervical cancer implementation research conforms to various levels of implementation science rigor. This allows the reader to readily identify papers most relevant to the various stages of implementation. The themes of this continuum are defined as follows: (1) Proof of Concept (Implementation not relevant or relevant, but not considered), (2) Proof of Implementation (Implementation relevant, but effects reduced), and (3) Informing Scale-up (Implementation studied as contributing factors or as primary focus) [17].

## Methods

We conducted a systematized literature review [18] of cervical cancer screening program implementation evaluations. Inclusion criteria were that the article (1) states the cervical cancer screening intervention occurred in a resource-limited setting in any country, (2) clearly defines implementation evaluation of the intervention with consideration of any defined implementation-relevant outcomes (acceptability, adoption, appropriateness, feasibility, fidelity, coverage, sustainability) [17], and (3) articulates evidence or intent of the intervention sustainment over time or through policy change by describing or reporting factors relevant to the inner (e.g., organizational characteristics, fidelity monitoring, staffing) or outer context (sociopolitical, funding, public-academic collaboration) [19]. The only exclusion criterion was publication in a language other than English.

### Search strategy

In July 2015, a search of PubMed, CINAHL, Web of Science, POPline, and IndMED included all years up to July 2015 in the initial title review and limited the search to publications in peer-reviewed journals. Search terms were based on four themes: cervical cancer screening, low resource, evaluation, and population level. (see additional file #1 for an example PubMed search string).

We used a multi-step process to select the articles for our review. Three independent reviewers conducted an initial screening for the inclusion criteria based on article titles and labeled them: “yes,” “no,” or “maybe.” If any of the reviewers labeled the title as a “yes” or “maybe,” it advanced to the next round of review. Articles identified as having a focus on cervical cancer were further screened to determine whether or not they included an evaluation of a screening intervention. Therefore, as a next step, abstracts and full articles (if needed) were reviewed by two reviewers to assess all inclusion and exclusion criteria. All discrepancies were discussed verbally in weekly meetings, with resolution by agreement between the two reviewers and an additional author. All data were managed through a shared and continually updated database.

### Data collection

The lead authors, through mutual agreement, devised and refined the data abstraction tool based, in part, on the Implementation research framework proposed by Peters et al. [17] Abstracted items included the following: location of study, partners, scale of intervention (national, regional, district, etc.), motivation of intervention, intervention description, study methodology, independent variables, screening approach (e.g., VIA, cytology, HPV), health system level of intervention, and identified implementation barriers. The two reviewers then each completed a test round of abstraction of the same 10 manuscripts. These abstracts were then reviewed and compared by one of the authors for discrepancies. The team met again to discuss these discrepancies and resolve any issues. The remaining articles were then abstracted by one of two reviewers. The team met once per week to discuss issues or questions related to abstraction.

Information was abstracted directly from the publication without interpretation for all categories with the following exceptions. To categorize the identified “Partners” (Table 1) and “Demand and Supply Side Implementation Barriers” (Table 2), we standardized the terminology with guidance from Peters [17] and Proctor [20]. To determine this categorization, both explicit information from the article as well as interpretation and commentary by the reviewing authors was considered. This subjective interpretation was necessary given the current lack of universal standardization of implementation science terminology [21]. Peters [17] includes guidance on the grouping of related terms, but it is not exhaustive and did not apply in all cases. Wherever subjective assessment was used, the lead author made a final determination on categorization for consistency. (Supplement 2).

**Table 1:** Description of Studies (*n* = 51)

	*N*	%	References
Years published			
≤ 2000	11	21.6	[25–35]
2001–2005	4	9.8	[36–39]
2006–2010	11	21.6	[13, 40–49]
2011–2015	25	49.0	[50–74]
Location by World Bank income level^a^			
High income	2	3.9	[25, 50]
Upper middle income	22	43.1	[26, 27, 29–31, 36, 37, 40, 42, 45, 46, 49, 51, 52, 54, 57–59, 63, 65, 69, 70]
Lower middle income	26	51.0	[13, 28, 32, 33, 35, 38, 39, 41, 43, 44, 47, 48, 55, 56, 59–62, 64, 66–68, 71–74]
Low income	5	9.8	[34, 53, 59, 61, 62]
Type of study design			
Experimental	7	13.7	[13, 36, 49, 50, 61, 66, 72]
Observational	17	33.3	[28, 29, 31, 40, 42, 45–48, 51, 52, 57, 65, 67, 70, 71, 74]
Descriptive	27	52.9	[25–27, 30, 32, 34, 35, 37–39, 41, 43, 44, 53–56, 58–60, 62–64, 68, 69, 73]
Type of primary screening technique^a^			
VIA	30	58.8	[13, 28, 31, 36, 39, 42–44, 46–49, 51–61, 63, 65–68, 72, 74]
VILI	8	15.7	[44, 48, 49, 52, 54, 57, 60, 72]
Cervicography	2	3.9	[36, 52]
Cytology (Pap)	20	39.2	[13, 25, 26, 28–35, 38, 41, 47, 48, 51, 52, 60, 61, 64]
HPV—self-collected	13	25.5	[27, 37, 40, 45, 52, 58, 61, 62, 67, 69–71, 73]
HPV—physician collected	9	17.6	[13, 31, 40, 44, 45, 52, 64, 67, 71]
Scale of intervention			
National	5	9.8	[42, 53, 62, 64, 65]
Regional	13	25.5	[25–28, 39, 40, 46, 54, 55, 59, 61, 68, 74]
Local	33	64.7	[13, 27, 29–36, 38, 43–45, 48–52, 57, 58, 60, 63, 66, 67, 69–73, 75]
Partners^a^ (*n* = 44)			
Academic institution—national	26	51.0	[26, 27, 29, 31, 32, 34, 36, 39, 40, 42–44, 46–48, 50–52, 54, 56, 57, 60, 67–69, 74]
Academic institution—international	19	37.3	[27, 28, 31, 36, 43–45, 47, 52, 55, 58, 60, 61, 64–66, 68, 71, 74]
Health system—local level	34	66.7	[13, 27, 29–36, 38, 43–45, 48–52, 55, 57, 58, 60, 63, 66, 67, 69–73, 75]
Health system—national	22	43.1	[13, 25, 26, 29, 30, 35, 38, 39, 42, 43, 49, 54, 55, 58, 61, 62, 64, 65, 68, 70, 71, 73]
Health system—international	3	5.9	[41, 52, 62]
NGO—local/national	9	17.6	[41, 46, 49, 53, 55, 60, 63, 71, 73]
NGO—international	19	37.3	[13, 29, 30, 39, 45, 47, 53, 55, 59–62, 64, 65, 68–71, 73]

VIA Visual Inspection with Acetic Acid, *VILI* Visual Inspection with Lugol's Iodine

^a^Categories are not mutually exclusive

**Table 2:** Implementation access level relevance

	*N*	%	Articles
Demand-side relevance^a^	44	86.3	
Patient/client-level	40	78.4	[13, 25–33, 35–38, 40, 42–45, 47, 49, 50, 53–55, 58–68, 70, 71, 73, 74]
Community engagement/outreach	30	58.9	[25, 27, 28, 30, 31, 35, 37, 38, 40, 41, 43, 47, 48, 50, 55, 58–61, 63–71, 74, 75]
Supply-side relevance^a^	35	68.6	
Human resources/provider capacity	30	58.9	[13, 25, 27, 28, 31, 33–35, 39–47, 49, 50, 54, 55, 58–60, 63, 65, 66, 68–70]
“Other”^b^ health system relevance	25	49.0	[13, 25, 27, 29–31, 34, 35, 39, 40, 42, 44, 46, 47, 49, 52, 59, 60, 65–70, 74]
Clinical services (quality)	13	25.5	[13, 27–29, 33, 35, 45–47, 59, 63, 65, 68]
Health system (policy)	12	23.5	[25, 34, 39, 44, 47, 49, 52, 60, 65, 67, 69, 74]
Both Demand and Supply-side relevance	31	60.8	[13, 25, 26, 28, 29, 31, 33, 35, 37, 40–45, 47, 49, 50, 54, 55, 58–60, 63, 65–70, 74]

Based on Levesque Patient-Centered Access to Healthcare Framework [22]

^a^Not mutually exclusive

^b^”Other” such as financing, information systems, equipment / resources management, leadership / governance

### Analysis and summary of findings

All lead authors were provided with the abstraction document and asked to independently assess emerging themes. Based on the abstracted information contained in the fields, motivation of intervention; description of intervention; methodology; variables; implementation barriers; findings; recommendations; and research or practice gaps were identified. We categorized the reviewed studies along a continuum of implementation research [17]. We assigned a single label to each paper among three categories: (1) Proof of Concept (Implementation not relevant or relevant, but not considered); (2) Proof of Implementation (Implementation relevant, but effects reduced); and (3) Informing Scale-up (Implementation studied as contributing factors or as primary focus). (Table 4).

Finally, we categorized barriers identified through the reported implementation research and organized them according to the Patient-Centered Access to Healthcare Framework proposed by Levesque [22]. This Framework specifies barriers on both the demand side (client and community perspective) as well as the supply side (health services level). Within this framework, demand-side barriers are subcategorized into the client’s ability to “Perceive,” “Seek,” “Reach,” “Pay,” and “Engage” with the community health system, while supply-side barriers are subcategorized into the health service’s “Approachability,” “Acceptability,” “Availability and Accommodation,” “Affordability,” and “Appropriateness” on the part of clients [22].

### Reporting

Reporting in this analysis refers to the Preferred Reporting Items for Systematic Reviews and Meta-Analyses (PRISMA) guidelines for reporting results of systematic reviews (S1) [23]. (Supplement 2).

## Results

Fifty-one of 13,330 initially identified papers met inclusion criteria. Twenty-five (49.0%) of the articles were published between 2011 and 2015 compared to only four (9.8%) being published between 2001 and 2005. When analyzed by the World Bank Income level [24], only five (9.8%) had sites discussed in the papers in low-income countries (three of which were in Uganda), while twenty-six (51.0%) sites were in lower-middle-income countries. (Table 1).

The studies reviewed and tested a variety of screening methods, and many of the studies tested multiple methods. Visual inspection was the most prevalent screening method, including 30 (58.8%) VIA studies and eight (15.7%) studies that considered VILI. Cytology testing was second most common, with 20 (39.2%) associated studies. The scale of intervention was distributed towards smaller-scale interventions. Thirty-three (64.7%) were conducted at the city or district level, while only five (9.8%) were at the national level. Forty-four of the 51 studies clearly described the involvement of multiple partners. Of all 51 studies, 26 (51.0%) indicated involvement of national academic institutions and 19 (37.3%) reported involvement of international institutions. Most engaged health systems including those at the local (*n* = 34, 66.7%), national (*n* = 22, 43%), and international (*n* = 3, 5.9%) levels.

For a more nuanced analysis of the reviewed articles, we categorized the relevance to various levels of implementation. (Table 2) We identified 40 (78.4%) articles pertinent to patient- or client-level considerations. Only 13 (25.5%) are applicable to the quality of clinical services. Twenty-five (49.0%) articles discussed additional health system-relevant themes (financing, information systems, equipment/resources management, leadership/governance, and others).

In addition to identifying the implementation access level of relevance, we, as well, sought to understand the explicit findings regarding barriers identified through the implementation research. Many of the papers reported specific findings relevant to demand-side (*n* = 26, 51.0%) or supply-side (*n* = 28, 49%) barriers to the successful implementation of cervical cancer screening services. From the articles presenting demand-side barriers (Table 3), those most often reported include comfort (*n* = 6, 23.1%), lack of knowledge (5, 19.2%), embarrassment associated with the clinical procedure (*n* = 3, 11.5%), and patient cost considerations (*n* = 3, 11.5%). Others receiving fewer mentions include distance to clinic, lack of patient priority for prevention, permission required by husband for procedure, and concern about no sexual intercourse after the procedure.

**Table 3:** Demand- and supply-side barriers (ranked by frequency)

Demand-side barriers	N	%	Supply-side barriers	*N*	%
	26^a^	51		28*	49
Multiple mentions			Provider-specific barriers	22^a^	78.6
Clinical procedure discomfort	6	23.1	Provider lack of opportunities/time for training	10	45.5
Client lack of knowledge	5	19.2	Provider shortages/turnover	9	41.0
Client embarrassment in the clinical setting	3	11.5	Trained provider having technical deficiency	6	27.3
Cost to client	3	11.5	Trained provider lack of counseling knowledge (psychosocial/resource availability/policy and guidelines awareness)	5	22.7
Distance to the clinic	2	7.7	Trained provider not offering service/competing priorities	3	13.6
Clients having low priority for prevention	2	7.7	Trained provider having technical approach bias	2	9.1
Permission required from husband	2	7.7			
Concern about no sexual intercourse after procedure	2	7.7			
Single mention			System-specific barriers	16^a^	57.1
Client concern about side effects	1	3.8	Cost to system	9	56.3
Screening is for promiscuous women	1	3.8	Lack of lab resources/malfunctioning equipment	7	43.8
Cervical Cancer is a curse	1	3.8	Facility distance to rural populations	4	25.0
Trust concerns with client-collected sample	1	3.8	Lack of supplies	3	18.8
Client concern about incorrect use of device	1	3.8	Lack of data-driven management	3	18.8
Challenges collecting sample in home environment	1	3.8	Lack of clinical space	2	12.5
Misperception that screening is not a primary concern or reason to visit the clinic (it is a secondary concern)	1	3.8	Lack of clinical supervision	1	6.3
Lack of immediate results	1	3.8	Ineffective referral systems/data management	1	6.3
Challenges with multiple visits or follow-up	1	3.8	Lack of policy/guidelines	1	6.3
Less acceptance in older women	1	3.8	Length of wait time/convenient appointment	1	6.3
Skepticism and Suspicion of the safety and efficacy of screening device, mainly among more educated women	1	3.8	Lack of electricity	1	6.3
Communication and language challenges	1	3.8			
Cultural barriers to diseases of reproductive system	1	3.8			
Lack of awareness of service availability	1	3.8			

^a^Categories are not mutually exclusive

Of the 28 papers reporting supply-side barriers (Table 3), 22 (78.6%) discuss provider-relevant themes and 16 (57.1%) discuss other health system-relevant considerations. Of the 22 reporting provider-associated barriers, 10 (45.5%) report a lack of opportunity or time to participate in trainings, nine (41.0%) report significant provider turnover as a major barrier, and 6 (27.3%) describe technical deficiencies of the provider as limitation to the impact of the screening program. Of the 16 reporting other system barriers, nine (56.3%) report cost as a major limiting factor in implementation.

As described in the background, we sought to characterize studies along the continuum of implementation research categories. (Table 4) In doing so, we illustrate the value of findings elucidated through studies with well-formulated implementation science questions. Twenty papers (39.2%) were labeled as Proof of Concept, 13 (25.4%) as Proof of Implementation, and 18 (35.3%) as Informing Scale-up. Table 5 details the publications categorized as informing scale-up, stratified by year.

**Table 4:** Categorization into the continuum of implementation [17]

	N	%	Articles
*Proof of Concept* Studies where implementation is not relevant or is relevant but not considered as research questions. The context of these studies is controlled and the factors affecting implementation are not relevant, fixed, or ignored Examples: Basic science, Phase I, II, & III clinical tr ials, efficacy studies, qualitative studies that are non-implementation or consider service quality	20	39.2	[13, 28, 31, 36, 39, 44–46, 48, 51, 52, 54, 56, 57, 60, 61, 64, 67, 72, 74]
*Proof of Implementation* Implementation variables are relevant but the effects are reduced. The context is real world with some control to intervention. Single implementation strategy. Implementation variables are equal or unchanging Examples: Pragmatic trials, Quasi-experimental, Observational studies with Implementation as secondary aim	13	25.5	[25, 27, 32, 33, 35, 38, 40, 42, 55, 63, 66, 71, 73]
*Informing Scale-up* Emphasize health system integration and sustainability as principal consideration. In these studies, implementation science contributed significantly or was the primary focus in the development of the research questions. Various methodologies may be used, such as participatory research, mixed methods, or observational studies, but implementation variables are either primary outcomes or independent variables Examples: Effectiveness implementation trials, participatory research, Mixed methods or quasi-experimental studies evaluating changes in delivery or acceptability, Observational studies with implementation as secondary factors or focused on adaptation, learning, and program scaling	18	35.3	[26, 29, 30, 34, 37, 41, 43, 47, 49, 50, 53, 58, 59, 62, 65, 68–70]

**Table 5:** Details of publications categorized as informing scale-up, stratified by year

Title	Authors	Year
Effect of a mobile unit on changes in knowledge and use of cervical cancer screening among rural Thai women	Swaddiwudhipong et al. [26]	1995
Evaluation of cervical cancer screening program in the Harare City Health Department, Zimbabwe	Moyo et al. [34]	1997
Evaluation of the cervical cancer screening program in Mexico: a population-based case–control study	Hernandez-Avile et al. [29]	1998
A mobile unit: an effective service for cervical cancer screening among rural Thai women	Swaddiwudhipong et al. [30]	1999
Experience with a self-administered device for cervical cancer screening by Thai women with different educational backgrounds	Sanchaisuriya et al. [37]	2004
A community-based education program about cervical cancer improves knowledge and screening behavior in Honduran women	Perkins et al. [41]	2007
Cervical cancer prevention: safety, acceptability, and feasibility of a single-visit approach in Accra, Ghana	Blumenthal et al. [43]	2007
Evaluation of cervical screening in rural North India	Bhatla et al. [47]	2009
A Three-year follow-up results of visual inspection with acetic acid/Lugol's iodine (VIA/VILI) used as an alternative screening method for cervical cancer in rural areas	Zhang et al. [49]	2010
A Promotora-administered group education intervention to promote breast and cervical cancer screening in a rural community along the U.S.-Mexico border: a randomized controlled trial	Nuño et al. [50]	2011
Acceptability of cervical cancer screening in rural Mozambique	Audet et al. [53]	2012
Feasibility of community-based careHPV for cervical cancer prevention in rural Thailand	Trope et al. [58]	2013
Screen-and-treat approach to cervical cancer prevention using visual inspection with acetic acid and cryotherapy: experiences, perceptions, and beliefs from demonstration projects in Peru, Uganda, and Vietnam	Paul et al. [59]	2013
Acceptability of self-collection sampling for HPV-DNA testing in low-resource settings: a mixed methods approach	Bansil et al. [62]	2014
Evaluation of a single-visit approach to cervical cancer screening and treatment in Guyana: feasibility, effectiveness and lessons learned	Martin et al. [65]	2014
Successes and challenges of establishing a cervical cancer screening and treatment program in western Kenya	Khozaim et al. [68]	2014
The development and evaluation of a community-based model for cervical cancer screening based on self-sampling	Belinson et al. [69]	2014
The Peru Cervical Cancer Screening Study (PERCAPS): the design and implementation of a mother/daughter screen, treat, and vaccinate program in the Peruvian jungle	Abuelo et al. [70]	2014

## Discussion

The number of articles (*n* = 51) evaluating the implementation of cervical cancer screening programs in limited resource settings is relatively small compared to the total number of articles identified through the review. Of note, we identified only five papers in low-income countries where settings are likely to have the greatest barriers to building sustainable capacity for cervical cancer screening and where, arguably, there is the most to be learned in vastly improving systems and approaches for screening. Of our reviewed articles, the majority (52.9%) were published between 2011 and 2015, suggesting an upward trend of reporting with the later time of publication. The reporting of experiences and sharing of best practices will contribute to our collective ability to overcome the many challenges in ensuring ultimate sustainability of these programs [76, 77].

The identified studies cover a range of cervical cancer screening methodologies with VIA being the most utilized and studied in our included papers. In addition, 46 (90.2%) papers describe research in decentralized settings with the majority of these (33 of 46, 71.7%) at the district or city level. As well, given the significant shortage of healthcare workforce globally [78], especially in resource-limited settings, it is not surprising that the overwhelming number of supply-side focused studies (32 of 37, 86.5%) considered capacity building. These findings may reflect a trend to integrate an effective, low-resource appropriate technology into existing health services in response to inequities in women’s health care and to strengthen primary health care in decentralized community health systems. The 2008 call [79] to offer more comprehensive packages of basic health services (including improved preventive care services) in all settings and more recent calls [12] to address non-communicable diseases (NCDs) are also consistent with this trend.

These findings provide some insight into the cervical cancer screening implementation literature. We note that articles commonly describe community- and client-relevant implications and explore challenges to human resources. Of interest, however, our analysis reveals that a relatively small percentage of papers describe or report quality assurance themes (25.5%) and a very low percentage (3.9%) describe quality improvement activities related to the implemented health service. Given that only 23.5% of all papers are describing “policy” in explicit terms, the present findings also illustrate a major gap in the literature regarding policy development around the long-term sustainment of cervical cancer screening programs.

Demand-side barriers are identified in 51% of reviewed articles with the most frequent focus on comfort, knowledge gaps, personal sensitivity, and cost. Provider issues (78.6%) make up most supply-side barriers. Much can also be learned from implementation evaluations describing other systems-level issues. The present analysis highlights other explicit concerns including cost, equipment, management practices, space, supervision, and infrastructure.

“Proof of Concept” papers describe studies where implementation is not relevant, or implementation is relevant but not considered as research questions. The context of these studies is focused and the factors affecting implementation are not relevant, fixed, or ignored. Because 20 studies fell into the “Proof of Concept” category and 13 in the “Proof of Implementation,” one could conclude that many of the published articles investigating cervical cancer screening programs are not implicitly structured to provide meaningful information of the real world context in which the research project occurs.

For example, a study in Western Kenya aimed to validate VILI as a stand-alone screening test at a Family AIDS Care and Education Services (FACES) clinic [72] while a study in Leon, Nicaragua compared the acceptability of self-collected versus clinician-collected human papillomavirus (HPV) tests which applies to the “proof of implementation” category [73].

“Proof of Concept” studies may be strengthened by examining more contextual factors to determine screening feasibility in similar settings. Implementation research also has the potential to describe in greater detail the supporting and hindering factors to wide-scale implementation and sustainment of a cervical cancer screening program within the context of their health system’s existing cancer control and prevention policy.

We classified 18 papers as “informing scale-up.” These papers provide useful guidance for developing cervical cancer implementation programs across different contexts. Principally, those contexts include in-depth perspectives on acceptability and community perceptions [53], community education and mobilization [30, 59] including radio messaging [41], community-focused or mobile screening [30, 58], detail on training community health workers [50], client tracking [59], maintenance of human capacity [59], task sharing [65], and quality control [70]. These findings are accessible and highly applicable to the existing programs struggling with substantial challenges as well as to institutions that are prioritizing the new implementation of cervical cancer screening services.A large study in 130 rural communities in Guangdong Province, China [69] employs sound Dissemination and Implementation research methods. Study results described community participatory research through the Chinese Cancer Prevention Study (CHICAPS). This program was conducted by community leaders with the technical assistance of the research team. They utilized a “pass the message on” model to easily reach women in communities through local village promoters that were trained through locally organized workshops (with up to 25 community leaders being oriented). Their paper describes the model process in depth, including details of stakeholder roles. Conclusions were that the model was successful in (1) improving the efficiency of resource utilization, (2) teaching community leaders and promoters to get patient information and follow procedures, and (3) teaching rural women technical specifics of the screening approach.

A three-phase evaluation of a cervical cancer screening program was conducted in the Harare city health department, Zimbabwe [34]. This study included a survey of policy makers on guidelines, policy, and attitudes regarding cervical cancer screening, and evaluating determinants at both the supply- and the demand side. Dissemination of their work provides invaluable guidance on how comprehensive policies on cervical cancer screening should be developed to assist in standardization of program implementation, how the formal technical training of health workers should be done, and what necessary resources should be allocated to support a successful and sustainable cervical cancer screening program.

Finally, a cervical cancer screening evaluation was conducted in Guyana to explore the feasibility, effectiveness, and lessons learned of a single-visit approach to cervical cancer screening and treatment [65]. The reported findings were highly relevant to sustainability and scale-up and concluded that certain components are essential to achieve good population coverage with high-quality services: (i) competency-based training and supportive supervision; (ii) task shifting to non-physician providers; (iii) a strong monitoring and evaluation system that rapidly identifies and addresses programmatic and clinical gaps; (iv) an enabling environment providing programmatic support; and (v) integration of cervical cancer prevention services into appropriate existing programs, such as family planning, postpartum, and HIV care.

### Limitations

Due to limitations in our search strategy and a lack of a risk of bias assessment, our review is characterized as a systematized review [18]. Given that our search strings were composed entirely of Medical Subject Heading (MESH) terms, we relied primarily on the accurate and current MESH terms and did not pursue the addition of articles through a free text search. Given the time limitations, MESH terms were used only to reduce the time associated with the search strategy development. This is problematic given that much of the published research from LMICs is not likely to be well indexed. Searches may therefore have left out relevant articles. Additionally, our search was conducted in the July of 2015. Any articles that were published after the search were not included in the review, omitting articles that would have met the criteria. The inclusion and exclusion criteria were strict for this literature review. Many articles were excluded from this review that provide valuable lessons in a variety of settings outside of the scope of our inclusion criteria, but were not regarded as employing implementation science. The findings from these excluded papers nevertheless may contain some findings relevant to implementation science and could be generalizable or be adaptable in areas with low resources. Additionally, it should be noted that the Continuum of Implementation Research as proposed by Peters [17] has limitations with a degree of subjectivity and author interpretation as described in the methods section. Every effort was made to consult the guiding framework and limit subjectivity by systematically and uniformly categorizing articles. Finally, the risk of bias for the included papers was not assessed.

## Conclusions

Many evidence-based health service interventions are not being readily adopted in LMICs because of an insufficient primary health care system in place to support them [79–81]. More Dissemination and Implementation research is needed to illustrate how health systems function at the local level, especially in LMICs [17]. Much of the existing Dissemination and Implementation science exploring the interface between health research and policy is concentrated in high-income countries. The paucity of similar research in LMICs presents a major challenge for implementation of preventive measures in these countries [82]. Given that cervical cancer can serve as a proxy for larger health systems issues, more detailed exploration of the barriers and best practices for increasing initial screening uptake and sustaining screening services over time may provide important insights to addressing other persisting women’s health issues and beyond, including the strengthening of broad primary health care services in low-resource settings. For programs wishing to move towards expanding the focus on their inquiry into this area, the two principal references that we have used to analyze the papers in this review are excellent resources for investigators new to implementation science [17, 22].

**Fig. 1:**
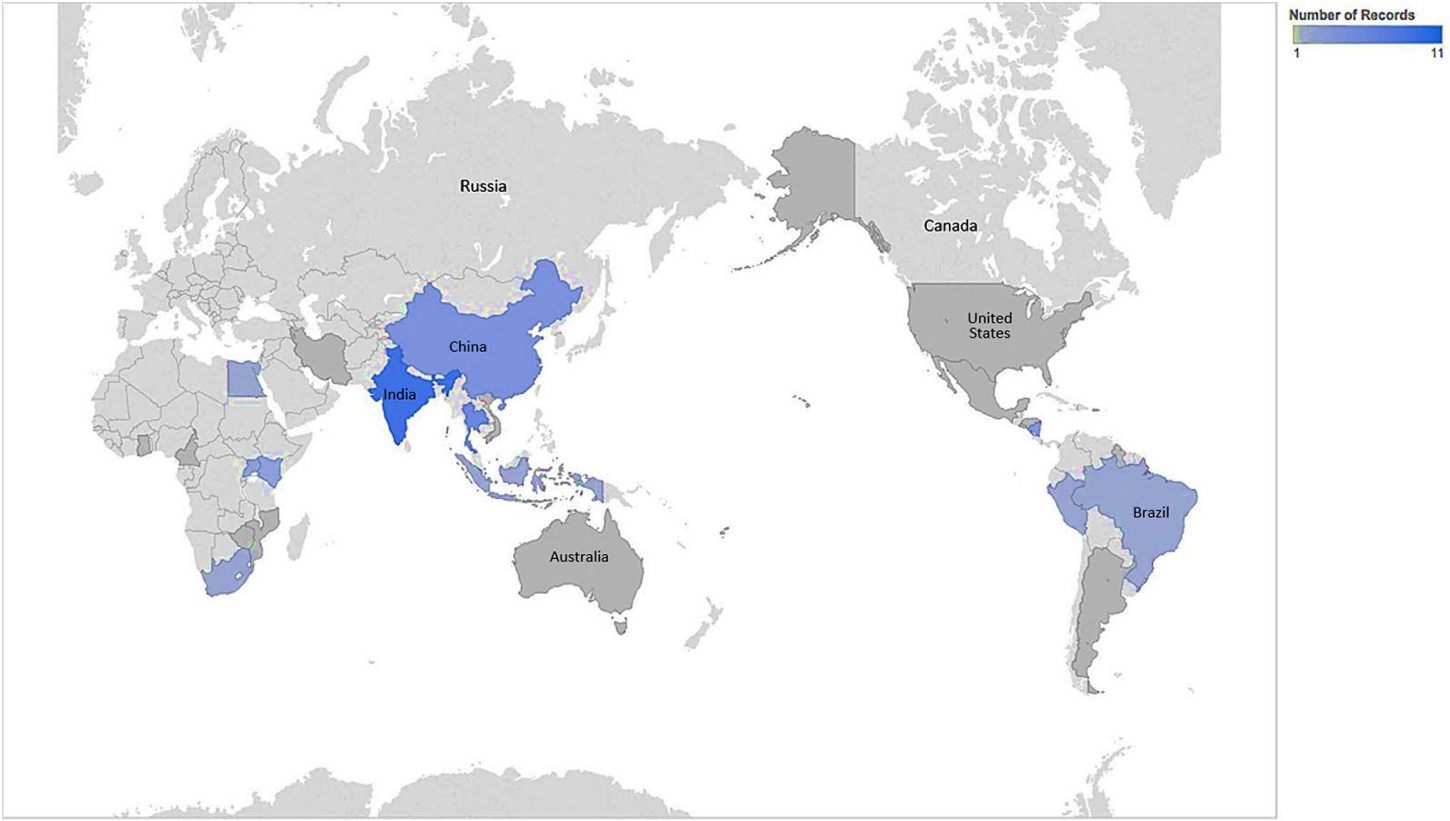
Country map of included articles

Given the overwhelming supporting evidence for the effectiveness of various screening technologies, it is unsettling that high cervical cancer incidence rates persist globally. There are clear downward trends of age-standardized incidence (ASI) rates in HIC, although no clear changes by period in low-income countries [83]. Given that a successful cervical cancer control and prevention program requires a robust systems approach including reliable access to primary healthcare, referral, and follow-up services, the incidence of cervical cancer has been shown to be an indicator for larger health systems issues [8]. Therefore, the implementation of cervical cancer prevention and control programs in areas with the least resources would have the greatest immediate impact on cervical cancer ASI rates while potentially favorably impacting other primary health care services. Unfortunately, there is a gradient between reviewed studies conducted in low-income countries (5, 9.8%) and LMICs (29, 56.9%), (Fig. 1). This disparity may be due to the profound difficulty of implementing cervical cancer programs and conducting research in states that are unstable or where infrastructure is significantly lacking. The implementation challenges in these settings may be the greatest to overcome in order to achieve sustainability of impactful interventions. These settings, unfortunately, may also possess the greatest challenges in conducting sound science, contributing to this well-documented research gap [84]. The areas with the greatest need for developing a clear understanding of implementation are, therefore, the most neglected. The dramatic lack of research from lower-resource environments to inform practice, in part, contributes to the continued gap in outcomes in such settings. However, these challenges could be opportunities for impact as well as for building knowledge. Replicating best practices from the most challenging contexts will likely lead to the greatest impact from dissemination of such scientific pursuits.

Program managers will benefit from working closely with researchers to report lessons learnt from programs implementing cervical cancer screening services. Furthermore, we urge researchers to move beyond technological innovation as the primary scientific pursuit and incorporate, when possible, implementation strategies to overcome barriers to health systems integration and sustainability. Researchers should evaluate the implementation of cervical cancer screening programs. This will build the science and practice of how to strengthen human resources capacity, develop responsive policy, and ensure sustained utilization of cervical cancer health services in different geographical settings.

## Electronic supplementary material

Below is the link to the electronic supplementary material.

Supplementary file1 (DOCX 12 kb)

Supplementary file2 (DOCX 48 kb)

Supplementary file3 (DOCX 17 kb)

## Data Availability

All materials, data, code, and associated protocols will be promptly available to readers without qualifications or restrictions. The datasets used and/or analyzed during the current study are available from the corresponding author on reasonable request.

## Abbreviations

ASI: Age-standardized incidence
CHICAPS: Chinese Cancer Prevention Study
D&I: Dissemination and implementation
FACES: Family AIDS Care and Education Services
HICs: High-income countries
HPV: Human papillomavirus
LEEP: Loop electrosurgical excision procedure
LMICs: Low- and middle-income countries
MESH: Medical subject heading
NCDs: Non-communicable diseases
NGO: Non-governmental organization
PERCAPS: Peru Cervical Cancer Screening Study
PRISMA: Preferred Reporting Items for Systematic Reviews and Meta-Analyses guidelines
VIA: Visual inspection with acetic acid
VILI: Visual inspection with Lugol's iodine
