# Supplementary material for: Evaluating the implementation of cervical cancer screening programs in low-resource settings globally: a systematized review
Source: Cancer Causes Control. 2020 Mar 17;31(5):417–29. doi: 10.1007/s10552-020-01290-4 (PMC7105425; doi:10.1007/s10552-020-01290-4)
Supplement: Supplementary file 1 — Supplementary file1 (DOCX 12 kb) [file 10552_2020_1290_MOESM1_ESM.docx]

## Supplemental Item #1 Example of PubMed search string

| *cervical cancer screening OR uterine cervical neoplasms OR mass screening OR HPV OR human papilloma virus OR "visual inspection" OR acetic acid/diagnostic use OR cytology OR vaginal smears OR papanicolaou test OR "pap smear" OR papillomaviridae/isolation and purification OR papillomaviridae infections OR cervical intraepithelial neoplasia OR early detection of cancer OR alphapapillomavirus/isolation and purification OR cervix uteri OR Lugol's iodine OR Lugol's solution) AND (developing countries OR "third world" OR socio-economic factors OR rural population OR "low resource" OR "low-resource") AND (Evaluation Studies as Topic OR Cost-benefit analysis OR Evaluation studies OR Program evaluation OR Health care evaluation mechanisms OR Health services research OR follow-up studies OR evaluat* [ti/ab]) AND population [ti/ab]* |
| --- |
