# Supplementary figures and images for: Evaluating the implementation of cervical cancer screening programs in low-resource settings globally: a systematized review
Source: Cancer Causes Control. 2020 Mar 17;31(5):417–29. doi: 10.1007/s10552-020-01290-4 (PMC7105425; doi:10.1007/s10552-020-01290-4)

## Supplemental Item #2 PRISMA flow diagram

##
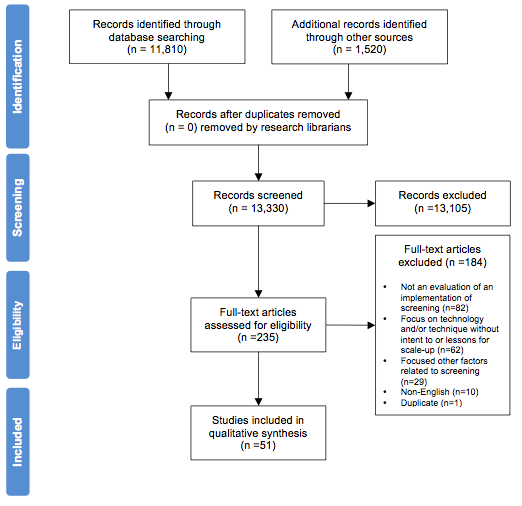

Supplement: Supplementary file 2 — Supplementary file2 (DOCX 48 kb) [file 10552_2020_1290_MOESM2_ESM.docx]
